# A tribute to Professor Yong Zhao

**DOI:** 10.1007/s13238-021-00875-2

**Published:** 2021-10-11

**Authors:** Zheng Tan, Jun Tang, Feng Wang, Xiaocui Li, Yanlian Chen, Zhou Songyang

**Affiliations:** 1 Institute of Zoology, Chinese Academy of Sciences (CAS), Beijing, 100101 China; 2 Xiamen Multi-Dimension Biomedical Technology Co., Ltd, Xiamen, 361000 China; 3 School of Basic Medical Sciences, Tianjin Medical University, Tianjin, 300203 China; 4 School of Life Sciences, Sun Yat-sen University, Guangzhou, 510275 China

Professor Yong Zhao was the Dean of School of Life Sciences at Sun Yat-sen University and a renowned biologist whose studies focused on the role of telomeres and telomerase in cancer and aging. Dr. Zhao’s postdoctoral mentor Professor Woodring E. Wright (1949–2019) of University of Texas Southwestern Medical Center (UTSW) once remarked, “It will be hard for anyone else in my lab to match up to the superb performance of Yong Zhao.”

**Figure 1:**
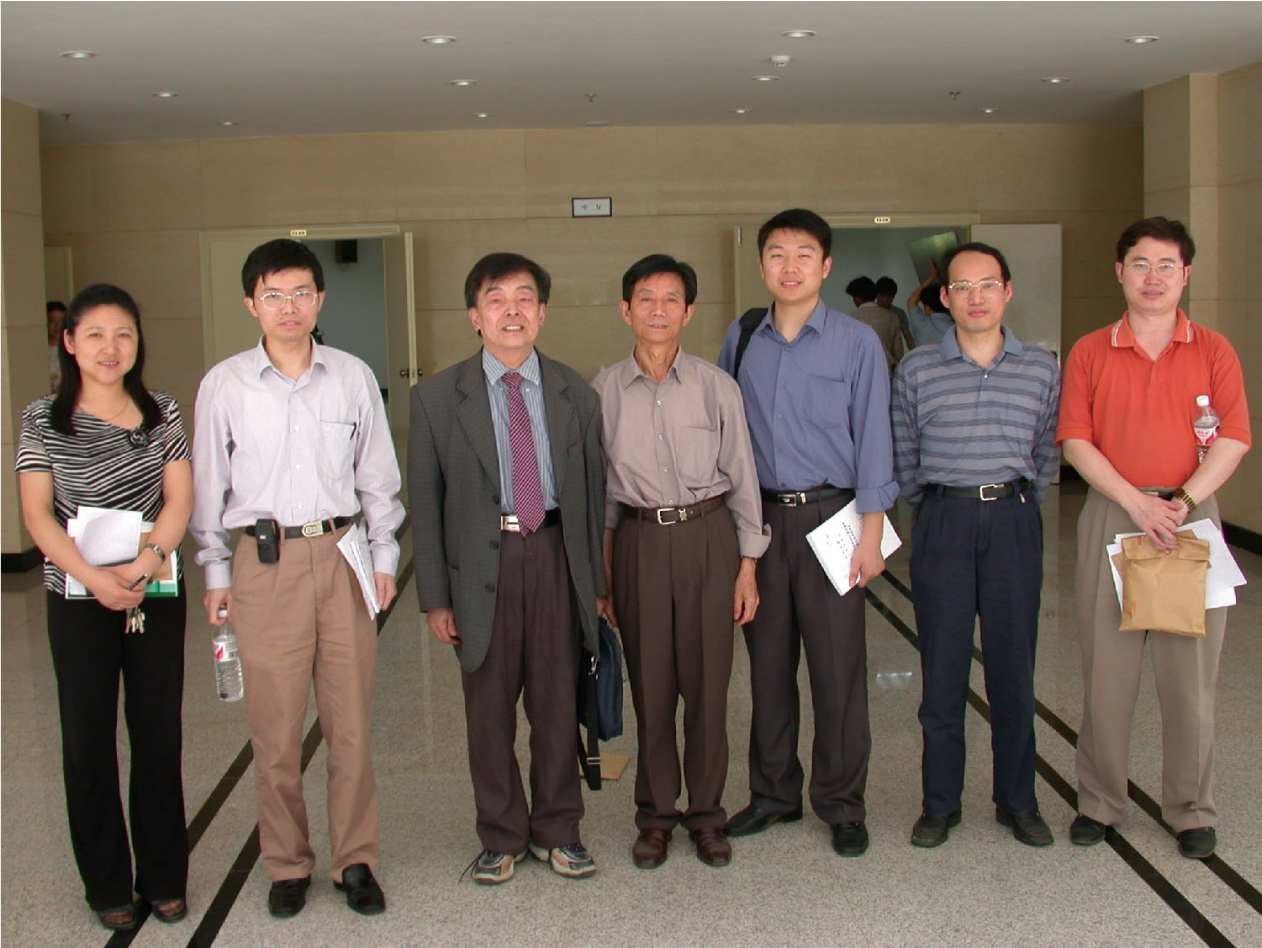
The doctoral graduation thesis defense of Yong in Wuhan

Yong was born on June 25th, 1976, in Dangyang, Hubei Province. He obtained his bachelor’s degree in Biochemistry from Nankai University in 1998 and his Doctor of Philosophy degree in Biophysics from College of Life Sciences at Wuhan University in 2003. For his doctoral thesis work under the guidance of Professor Zheng Tan (Fig. 1), Yong developed a method that enabled simultaneous and quantitative measurements of multiple kinetic parameters during nucleic acid duplex formation using an optical biosensor based on surface plasmon resonance. When applied to the study of human telomere G-quadruplexes, it revealed the dynamic structure of human telomeres and helped expand the application of optical biosensors (Zhao et al., 2004).

**Figure 2:**
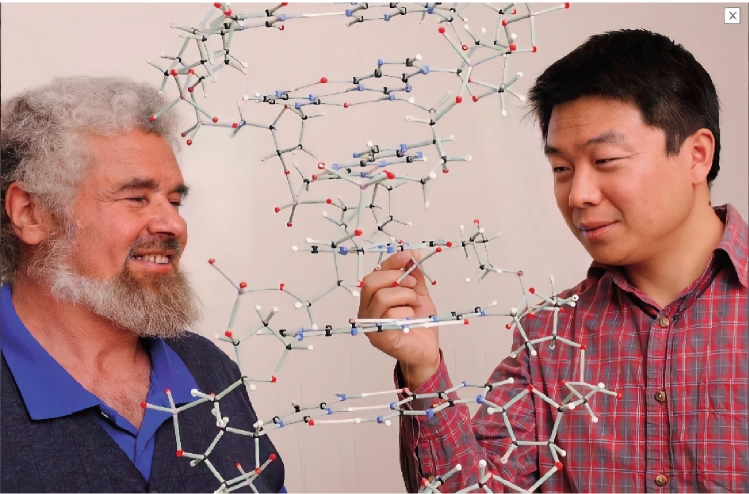
Professor Woodring E. Wright and Yong in USA

After completing his postdoctoral research in analytical chemistry at the College of Chemistry and Molecular Sciences in Wuhan University in 2006, Yong continued his postdoctoral training in cell biology under the tutelage of Professors Woodring E. Wright and Jerry W. Shay at UTSW (Fig. 2), where his ingenuity, creativity, and tenacity shone again. For example, he developed new ways to quantify the length of telomeric overhangs (Zhao et al., 2008), and monitor the processes of telomeric G-strand extension and C-strand fill-in (Zhao et al., 2009). Using these innovative methods, Yong provided concrete evidence that had led to the revision of the telomere elongation model to a stepwise process where G-strand extension during the S phase is followed by C-strand fill-in during the late S/G_2_ phase. This outstanding discovery, published as a Feature Article in the leading science journal *Cell* (Zhao et al., 2009), also provides additional targets for the development of anti-cancer therapeutics. In 2009, Yong joined the UTSW faculty as an Assistant Lecturer, where his research has helped refine the understanding of telomerase-dependent elongation of telomeres. For example, his work revealed the dichotomy in telomerase action in which multiple telomerase molecules can act on each telomere end under non-equilibrium conditions as opposed to a single telomerase molecule at each telomere end under steady-state conditions. These results were published as the cover story in the journal *Molecular Cell* (Zhao et al., 2011).

**Figure 3:**
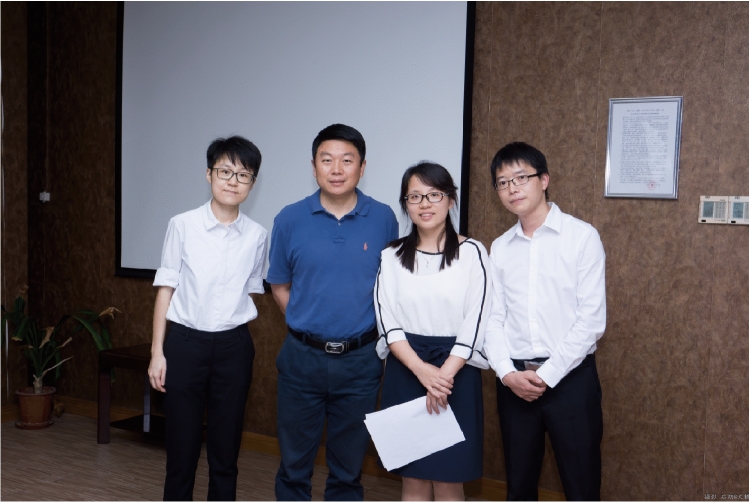
Yong and his doctoral students in Guangzhou

In the summer of 2011, Yong returned to China and devoted his life to scientific research in his homeland. He was appointed as 100 Top Talents Professor in the School of Life Sciences at Sun Yat-sen University and established the Laboratory of Cell Aging and Cancer Research in Guangzhou (Fig. 3). It was here that he led his students to the discovery that telomeric double-strand breaks (DSBs) can be efficiently repaired by homologous recombination in proliferating but not stress-induced or replicative senescent cells, contrary to the then widely held belief that telomeric DSBs could not be repaired in cells and would thus trigger cell senescence (Mao et al., 2016). Professor Zhao’s group also found p53 and AKT to be key factors in suppressing spontaneous apoptosis in ALT cells, which points to the inhibition of p53 and AKT as a new therapeutic approach that specifically targets ALT cancers (Ge et al., 2019). With the unprecedented spread of the novel coronavirus SARS-CoV-2 since the end of 2019, COVID-19 has quickly become a global health issue. Yong and his team were able to identify the small molecule C1632 as an inhibitor that could block SARS-CoV-2 replication and viral-induced inflammation by upregulating the *let-7* gene (Xie et al., 2021). This exciting work underlines C1632 as a potential therapeutic candidate in treating COVID-19.

Professor Yong Zhao garnered national and international attention for his work in the cancer and aging fields, with over 40 published articles and book chapters as well as 6 patents in the areas of telomere analysis and anti-cancer therapies. For his outstanding work and achievements, Yong received many honors. He was awarded postdoctoral fellowships by the American Federation for Aging Research and the Ellison Medical Foundation. And he was the key member in the Innovative Scientific Research Team of Guangdong Province to develop targeted anticancer drugs and vaccines. Yong won the Excellent Young Scientists Fund in 2013 and was named a Pearl River Scholar of Guangdong Province in the same year. His significant contributions earned him the National Science Fund for Distinguished Young Scholars in 2020.

Yong was above all a consummate educator. Serving as Party Secretary (2018) and then Dean (2020) of the School of Life Sciences, he wholeheartedly devoted his time and energy to improving the quality of teaching and motivating student learning. He was particularly generous with his time for students, often meeting with them face to face to provide support and encouragement. Unfailingly kind and dedicated, Yong went far beyond merely teaching techniques and knowledge, he was committed to fostering collaborative spirits and critical thinking skills in all the students.

The sudden and unexpected passing of Professor Yong Zhao at the age of 45 on April 8th, 2021 has left a huge void. A compassionate patriot, a distinguished scientist, an advocate for science and education, a skillful leader of the School of Life Sciences, a respected and big-hearted colleague, and an inspiring and beloved teacher, Yong had touched so many lives in so many ways. He will be dearly missed and forever present in the hearts and memories of everyone lucky enough to have known him.
